# Osteogenic differentiation of bone marrow stromal cells is hindered by the presence of intervertebral disc cells

**DOI:** 10.1186/s13075-015-0900-2

**Published:** 2015-12-25

**Authors:** Samantha C. W. Chan, Adel Tekari, Lorin M. Benneker, Paul F. Heini, Benjamin Gantenbein

**Affiliations:** Tissue and Organ Mechanobiology, Institute for Surgical Technology and Biomechanics, University of Bern, Stauffacherstrasse 78, Bern, CH-3014 Switzerland; Biointerfaces, EMPA, Swiss Federal Laboratories for Materials Science and Technology, Lerchenfeldstrasse 5, St Gallen, CH-9014 Switzerland; Department for Orthopedic Surgery and Traumatology, Inselspital, University of Bern, Freiburgstrasse 4, Bern, CH-3010 Switzerland; AOSpine Research Network, Stettbachstrasse 6, Dübendorf, CH-8600 Switzerland; Orthopedic Department, Sonnenhof Clinic, Buchserstrasse 30, Bern, CH-3006 Switzerland

**Keywords:** Spinal non-fusion, Osteogenesis, Bone marrow, Mesenchymal stem cells, Human nucleus pulposus cells, Human annulus fibrosus cells, Relative gene expression

## Abstract

**Background:**

Clinical observations indicate that the presence of nucleus pulposus (NP) tissue during spinal fusion hinders the rate of disc ossification. While the underlying mechanism remains unknown, this observation could be due to incomplete removal of NP cells (NPCs) that secrete factors preventing disc calcification, such as bone morphogenetic protein (BMP) antagonists including noggin and members of the DAN (differential screening selected gene aberrative in neuroblastoma) family.

**Methods:**

Monolayer human bone marrow-derived mesenchymal stem cells (MSCs) were cocultured withNPCs and annulus fibrosus cells (AFCs) embedded in alginate for 21 days. At the end of coculture, MSCs were stained for mineral deposition by alizarin red, and relative expression of bone-related genes [*Runt-related transcription factor 2, (RUNX2), Osteopontin (OPN)*, and *Alkaline phosphatase (ALP)*] and ALP activity were analyzed. Relative expression of three BMP antagonists, *chordin* (*CHRD*), *gremlin* (*GREM1*), and *noggin* (*NOG*), was determined in primary human NPCs and AFCs. These cells were also stained for Gremlin and Noggin by immunocytochemistry.

**Results:**

Alizarin red staining showed that MSC osteogenesis in monolayer cultures was inhibited by coculture with NPCs or AFCs. ALP activity and RT-PCR analyses confirmed these results and demonstrated inhibition of osteogenesis of MSC in the presence of disc cells. NOG was significantly up-regulated in MSCs after coculture. Relative gene expression of intervertebral disc (IVD) cells showed higher expression of *GREM1* in NPCs than in AFCs.

**Conclusions:**

We show that primary IVD cells inhibit osteogenesis of MSCs. BMP inhibitors *NOG*, *GREM1* and *CHRD* were expressed in IVD cells. *GREM1* appears to be differentially expressed in NPCs and AFCs. Our results have implications for the design and development of treatments for non-union in spinal fusion.

## Background

Spinal fusion is a surgical procedure to join two or more vertebral bodies together by removal of the intervertebral disc (IVD). During this process, the disc is dissected and a spinal cage is placed in the disc space together with either an allogeneic or autogenic bone graft or a synthetic bone substitute with the support of growth factors. To obtain segmental stability, the procedure relies on the natural bone growth processes of the spine. Although excision of an IVD is a standard procedure for spinal fusion, the development of minimally invasive operations, such as laparoscopic anterior spinal fusion with cages, may result in incomplete discectomy.

Based on a retrospective study, Lee et al*.* [1] and Watkins et al. [2] reported non-fusion rates ranging from 0–30 %. Based on clinical observations, the presence of nucleus pulposus (NP) tissue appears to hinder the rate of disc ossification after spinal fusion surgery [3]. Further evidence supporting inhibition of ossification was gained in an animal study performed on pigs, which also suggested a delay in bone formation during spinal fusion in the presence of NP tissue [4]. By understanding the underlying mechanism of this process, strategies to enhance spinal fusion can be investigated.

The NP is derived from the notochord of mesodermal origin [5]. During development, the notochord in the human embryo gradually breaks down and is enclosed within the primitive annulus fibrosus (AF) that later forms the primitive NP [6]. Noggin (*NOG*) and sonic hedgehog are expressed during notochord development [7]. IVD cells secrete bone morphogenetic protein (BMP)-binding proteins, such as *NOG*, gremlin (*GREM*1), and chordin (*CHRD*), to block BMP signaling that originates from the vertebral bodies to provide a BMP-reduced zone and prevent ossification of the disc [8, 9]. The expression of these BMP antagonists in IVD cells is important for normal formation of the disc and plays a role in its segmental pattern formation [7, 10]. BMPs, which are members of the transforming growth factor-β superfamily, play a pivotal role in skeletal development because they induce commitment of mesenchymal stem cells (MSCs) toward osteoblasts [11–15]. BMP signals are regulated by extracellular and intracellular signals through BMP antagonists that block BMP signal transduction at multiple levels [16]. By examining notochord-specific markers, such as *brachyury*, a previous study has shown that notochordal cells are present in human discs throughout life [5]. It is therefore possible that nucleus pulposus cells (NPCs) also express certain BMP antagonists. *CHRD* has also been reported to be expressed in both bovine and human IVD tissues [17]. Otherwise, there is limited information on the expression of the BMP antagonists in human IVDs.

The balance between bone, cartilage, and IVDs in skeletal development and its homeostasis are critical to understanding the development of disease [16, 18, 19]. It is thus important to investigate the reported BMP antagonists that are likely secreted by IVD cells and might influence the osteogenic differentiation of MSCs and functions of osteoblasts to improve spinal fusion efficacy. This issue is significant considering a study on the application of high doses of BMP2, which queried the safe and efficient application of such cytokines in the clinic [20]. It has been recently shown that specific members of the differential screening selected gene aberrative in neuroblastoma (DAN) family [18] and BMP antagonists such as Noggin might be responsible for delaying and inhibiting spinal fusion, although such cytokines are applied at very high doses [16, 21]. Therefore, we hypothesized that NP cells secret cytokines [22, 23] that affect the metabolism of osteoblasts or bone marrow-derived MSCs [24]. Here, we investigated the effect of IVD cells on osteogenesis of MSCs and provide fundamental information on which bone-forming inhibitors are secreted by IVD cells to reveal the mechanism of delayed ossification in the presence of NP tissue during spinal fusion. The first aim was to investigate the role of NPCs and annulus fibrosus cells (AFCs) on the osteogenesis of MSCs. The second aim was to analyze the natural expression of candidate BMP inhibitors. To this end, three BMP antagonists, *CHRD*, *GREM1* and *NOG*, were evaluated at mRNA and protein levels in enzyme-isolated, low passage-expanded human IVD cells using gene expression and immunocytochemical analyses.

## Methods

### Human donor materials

Human bone marrow and IVD tissues were collected from patients who underwent spinal surgery. Written informed consent was obtained from the patients. The procedure was approved by Ethics Office of the Canton of Bern. The donors of MSC and IVD samples used for the coculture experiment are listed in Table 1. MSCs were isolated from five patients, and IVD cells were isolated from six patients. Three discs were symptomatic degenerative discs (SD), two discs were discs affected by trauma (T), and one patient was found to have an asymptomatic degenerative disc (D,T). IVD materials from another eight human donors were used to isolate disc cells for immunocytochemistry and measure the mRNA levels of BMP antagonists, which are listed in Table 2. Four discs were symptomatic degenerative discs, two discs were trauma discs and discs from two patients were asymptomatic degenerative discs.

**Table 1:** Donors of mesenchymal stem cells (MSCs) and intervertebral disc (IVD) materials for coculture experiments

	Bone marrow-derived MSCs				IVD cells						
Coculture experiment	MSC donor	Birth year	Patient age, years	Sex	IVD donor	Disc level	Grade of disc degeneration	Birth year	Patient age, years	Sex	Type
1	1	1932	81	F	1	L1–L2	Unknown	1988	25	M	T
2	2	1951	62	F	1	L1–L2	Unknown	1988	25	M	T
3	3	1958	55	M	2	L1–L2	Pfirrmann 2 (CT)	1975	38	M	D, T
4	3	1958	55	M	3	L4–L5	Pfirrmann 3 (CT)	1957	56	M	SD
5	4	1957	57	F	2	L1–L2	Pfirrmann 2 (CT)	1975	38	M	D, T
6	4	1957	57	F	4	L4–L5	Pfirrmann 3 (CT)	1957	56	M	SD
7	4	1957	57	F	5	L1–L2	Pfirrmann 1–2 (MRI)	1978	35	M	T
8	5	1989	25	M	6	T12–L1	Unknown	1978	36	M	SD

MSCs were isolated from five patients and IVD cells were isolated from six patients. *F* female, *M* male, *L* lumbar vertebra, *CT* computed tomography, *MRI* magnetic resonance imaging, *T* traumatic disc, *D* degenerative disc, *SD* symptomatic degenerative disc

**Table 2:** Donors of IVD materials for cell isolation, and RT-PCR and immunostaining analyses of bone morphogenic protein antagonists

IVD donor	Donor code	Birth year	Sex	Patient age, years	Disc level	Type
1	P38	1978	M	37	L5–S1	SD, modic type II
2	P40	1959	M	56	L4–L5	SD, modic type II
3	P41	1982	M	33	Unknown	T
4	P45	1968	M	47	T12–L1	SD
5	P46	1968	M	47	L1–L2	T, D
6	P47	1982	M	33	T12–L1	T,D
7	P48	1941	M	74	L2–L3	T
8	P50	1958	M	57	L5–S1	SD

*M* male, *L* lumbar vertebra, *T* traumatic disc, *D* degenerative disc, *SD* symptomatic degenerative disc

### Human cell isolation and expansion

Mononuclear cells from bone marrow samples were isolated by density gradient centrifugation (Histopaque-1077, Sigma-Aldrich, Buchs, Switzerland) and then plated in minimum essential medium alpha (α-MEM) containing 10 % fetal bovine serum (FBS, Sigma-Aldrich), 100 U/ml penicillin, 100 mg/ml streptomycin, and 2.5 ng/ml fibroblast growth factor 2 (PeproTech, London, UK). The MSCs were expanded as monolayers up to passage three to homogenize the cell populations and increase the cell number. IVD tissues were processed within 24 h after isolation and divided into the NP and AF by an experienced surgeon. Cells from both tissues were separated from their native surrounding extracellular matrix by digestion with 0.19 % pronase (Roche, Basel, Switzerland) for 1 h and then collagenase type 2 (Worthington, London, UK) overnight (approximately 14 h) as described previously [25]. The disc cells were expanded in low glucose (1 g/L) Dulbecco’s modified Eagle’s medium (LG-DMEM) containing 10 % FBS, 100 U/ml penicillin, and 100 mg/ml streptomycin. Both cells types were expanded up to passage 2 prior to coculture experiments (Table 1).

### Cell encapsulation and coculture

**Fig. 1:**
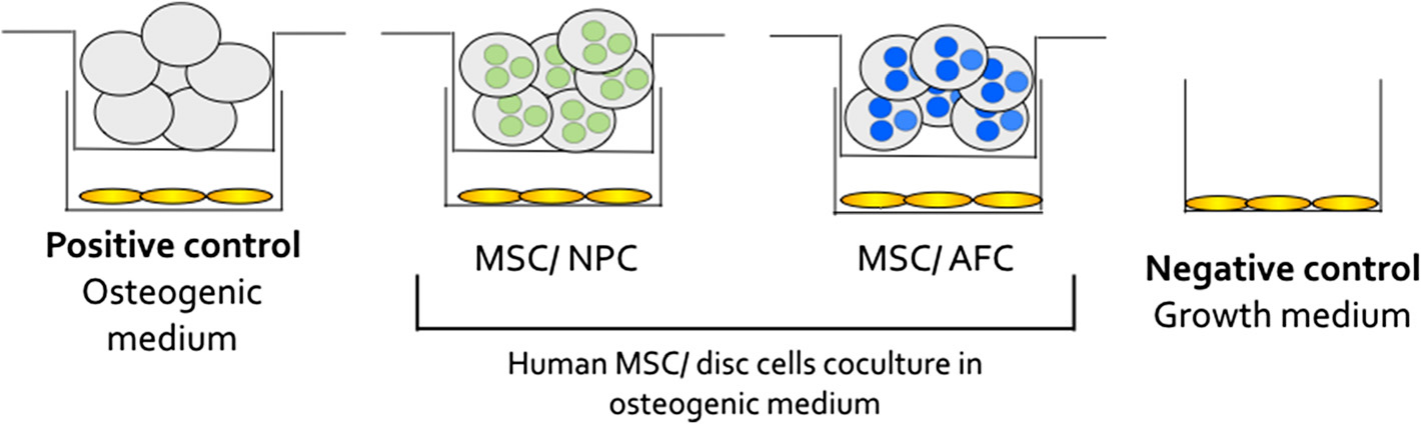
Design of coculture experiments with human bone marrow-derived mesenchymal stem cells (MSCs) and intervertebral disc cells without cell contact. Four experimental groups were cultured for 21 days. Controls were MSCs cultured in osteogenic medium (positive control) and MSCs cultured in growth medium (negative control). The experimental groups were cocultures of MSCs with either nucleus pulposus cells (NPCs) or annulus fibrosus cells (AFCs)

MSCs were seeded at 2 × 10^4^ cells/well in 12-well plates. NPCs and AFCs were encapsulated separately in 1.2 % alginate at a density of 4 Mio/mL using a syringe (22 G needle) by forming approximately 30 μl droplets in a 102 mM CaCl_2_ salt solution [26]. The NPC and AFC alginate beads (six beads/well) were maintained in coculture with a monolayer of MSCs in osteogenic medium (α-MEM) containing 10 % FBS, 100 U/ml penicillin, 100 mg/ml streptomycin, 10 nM dexamethasone, 5 mM β-glycerophosphate, and 50 μg/ml ascorbic acid-2-phosphate (all purchased from Sigma-Aldrich). MSC coculture with empty alginate beads served as a positive material control (Fig. 1). The negative control was a MSC monolayer cultured in growth medium without osteogenic supplements. All cocultures were conducted using culture inserts (0.4-μm pore size, high pore density, polyethylene terephthalate track-etched, Becton, Dickinson and Company, Allschwil, Switzerland) to ensure diffusion of cytokines and no direct cell contact. The cultures were maintained for 21 days with medium changes three times per week.

### Real-time RT-PCR

Relative gene expression of major osteogenic genes, including *Alkaline Phosphatase* (*ALP*), *Runt-related transcription factor 2 (RUNX2)*, and *Osteopontin* (*OPN*)*,* BMP antagonists *NOG* and *GREM1*, and ribosomal *18S* as a reference gene [27] were monitored in MSCs. To determine the baseline expression levels of BMP antagonists in disc cells, *CHRD*, *NOG* and *GREM1* gene expression was monitored in passage-1 primary IVD cells. Cells were isolated from the extracellular matrix by enzymatic digestion and then cultured as a monolayer. This approach was chosen over bead culture to minimize artifacts from extended in vitro culture. Human-specific oligonucleotide primers (Table 3) (Microsynth, Balgach, Switzerland) were designed with Beacon Designer™ software (Premier Biosoft, Palo Alto, CA, USA) based on nucleotide sequences from GenBank. Amplicons were generated using a two-step protocol using 61 °C for 30 s and melting at 95 °C for 15 s with 45 cycles. Relative gene expression was quantified by application of a threshold cycle (C_t_) and calculation of ∆∆C_t_. The statistical analysis of 2^-∆∆Ct^ was conducted according to Livak and Schmittgen [28].

**Table 3:** Primer sequences for real-time PCR analyses

Gene	Forward primer	Reverse primer
*18S*	CGA TGC GGC GGC GTT ATT C	TCT GTC AAT CCT GTC CGT GTC C
*Alkaline phosphatase (ALP)*	GTA TGA GAG TGA CGA GAA	AAT AGG TAG TCC ACA TTG T
*Runt-related transcription factor 2 (RUNX2)*	AGC AGC ACT CCA TAT CTC T	TTC CAT CAG CGT CAA CAC
*Osteopontin (OPN)*	ACG CCG ACC AAG GAA AAC TC	GTC CAT AAA CCA CAC TATC ACC TCG
*Noggin (NOG)*	CAG CAC TAT CTC CAC ATC CG	CAG CAG CGT CTC GTT CAG
*Gremlin (GREM1)*	GAG AAG ACG ACG AGA GTA AGG AA	CCA ACC AGT AGC AGA TGA ACA G
*Chordin (CHRD)*	GTG GCT GCT GTG ACT CTG	AAC AGG ACA CTG CCA TTG

### Alkaline phosphatase (ALP) activity

ALP activity was quantitatively measured using a commercial phosphatase assay kit (Sigma-Aldrich) according to the manufacturer’s instructions. The cells were lysed with celLytic M (Sigma-Aldrich) and freeze-thawed at −20 °C and room temperature twice to release the ALP. The lysate was transferred to 96-well plates and incubated with ALP substrate at 37 °C for 30 minutes. The reaction was stopped by addition of the stop buffer. The p-nitrophenol product formed by enzymatic hydrolysis of the p-nitrophenyl phosphate substrate was measured at 405 nm using a microplate reader (SpectraMax M5, Bucher Biotec, Basel, Switzerland).

### Histological staining of cell mineralization

To visualize the amount of calcium deposition by MSCs after 21 days of culture under osteogenic-inducing conditions, the cells were fixed in 4 % formalin for 10 minutes and then stained with a 2 % Alizarin red S solution (Sigma-Aldrich) for 45 minutes. The cells were washed with distilled water three times and then once with phosphate-buffered saline (PBS). High-resolution images were then captured with a digital camera (Eclipse 800, Nikon, Tokyo, Japan).

### Immunocytochemical staining and detection of GREM1 and NOG in primary IVD cells

Passage-1 human NPCs and AFCs were grown on glass coverslips for 48 h in 6-well plates at a density of 5 × 10^4^ cells/well in LG-DMEM containing 10 % FBS, 100 U/ml penicillin, and 100 mg/ml streptomycin. The cells were then fixed with 4 % buffered paraformaldehyde for 10 minutes and stored in PBS at 4 °C prior to staining. Cells were then permeabilized with 100 % methanol for 2 minutes, rehydrated in PBS for 2 minutes, blocked with 10 % FBS in PBS for 1 h, and then incubated with primary antibodies (1:200 dilutions) at 4 °C overnight. Antibodies were rabbit anti-Gremlin-1 (sc-28873, Santa Cruz Biotechnology Inc. Dallas, TX, US) and mouse anti-Noggin (SAB3300029, Sigma-Aldrich) monoclonal antibodies for double immunostaining. For the staining control, primary antibodies were omitted. Secondary antibodies were added at 1:1000 dilutions and incubated for 1 h at room temperature (A-11008 Alexa Fluor 488 goat anti-rabbit and Alexa Fluor 555 goat anti-mouse IGg2b (y2b), Molecular Probes, Life Technologies, Basel, Switzerland). The cells were visualized with a confocal laser scanning microscope (cLSM710, Carl Zeiss, Jena, Germany) at × 63 magnification.

### Statistics

Statistical analysis was performed using PRISM software (version 6.0f, GraphPad, La Jolla, CA, USA). A value of *p* <0.05 was considered to be significant. Ordinary one-way analysis of variance with Tukey’s multiple comparison test was applied for all analyses.

## Results

### Histology of calcium deposition

**Fig. 2:**
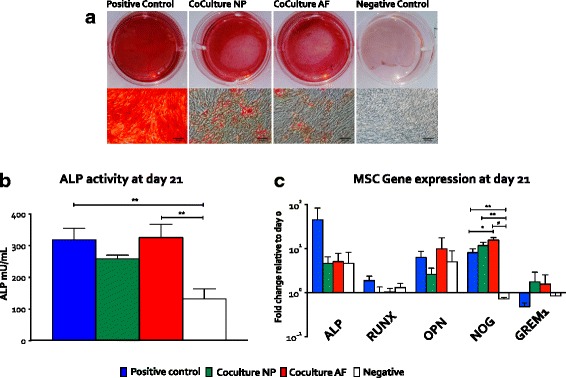
Osteogenic differentiation of bone marrow-derived mesenchymal stem cells (MSCs) in coculture with nucleus pulposus cells (NPCs) and annulus fibrosus cells (AFCs). **a** Alizarin red staining. Macroscopic (*top row*) and microscopic images (*bottom row,* ×10 magnification) showed reduced alizarin staining of MSCs in coculture with NPCs or AFCs compared with the positive control. *Scale bar* 100 μm. **b** Alkaline phosphatase (ALP) activity of MSCs after 21 days (n = 5) showed an increase under all osteogenic conditions compared with the negative control. ALP activity was reduced in cocultures of MSCs with NPCs and similar levels were found between cocultures of MSCs with AFCs and the positive control. **c** Relative gene expression of osteogenic markers (*ALP*, *RUNX2* and *OPN*) in MSCs in response to the presence of intervertebral disc (IVD) cells in alginate beads (n = 5). Cocultures with IVD cells showed reduced gene expression of these osteogenic markers compared with the positive control. Furthermore, a significant increase in gene expression of *NOG* was observed under the osteogenic condition: *p <0.05, **p <0.01, ***p <0.001, and ^#^ *p* <0.0001

MSCs grown in osteogenic medium had the greatest calcium deposition (Fig. 2a), which served as the positive control. Coculture of MSCs and IVD cells resulted in a reduction of mineralization as observed by reduced alizarin red staining. These results were comparable with MSCs cultured in growth medium (negative control).

### ALP activity in MSCs

After 21 days of culture, ALP activity was increased in MSCs cultured in osteogenic medium compared with the negative control (Fig. 2b). ALP activity of the positive control was 318.7 ± 35.71 mU/ml, which differed significantly from that of the negative control (132.0 ± 31.84 mU/ml, *p* = 0.0045). MSC-NPC cocultures did not differ from the negative control (*p* = 0.061). However, MSC-AFC cocultures differed significantly from the negative control (*p* = 0.003).

### Relative gene expression of bone-related markers in MSCs

Relative gene expression analysis of bone-related markers showed higher *ALP, Runx2*, and *OPN* expression in the positive control than that in the negative control (Fig. 2c). Gene expression of *ALP*, *Runx2*, and *OPN* in cocultures was comparable with the negative control. *NOG* gene expression was significantly increased in osteogenic cultures compared with the negative control (*p =* 0.042, negative vs positive controls; *p =* 0.001, negative control vs MSC-NPC cocultures; *p* >0.0001, negative control vs MSC-AFC cocultures). Gene expression of *NOG* in MSCs was also higher in MSC-AFC cocultures than in the positive control (*p* = 0.037).

### Protein expression of NOG and GREM1 in IVD cells

**Fig. 3:**
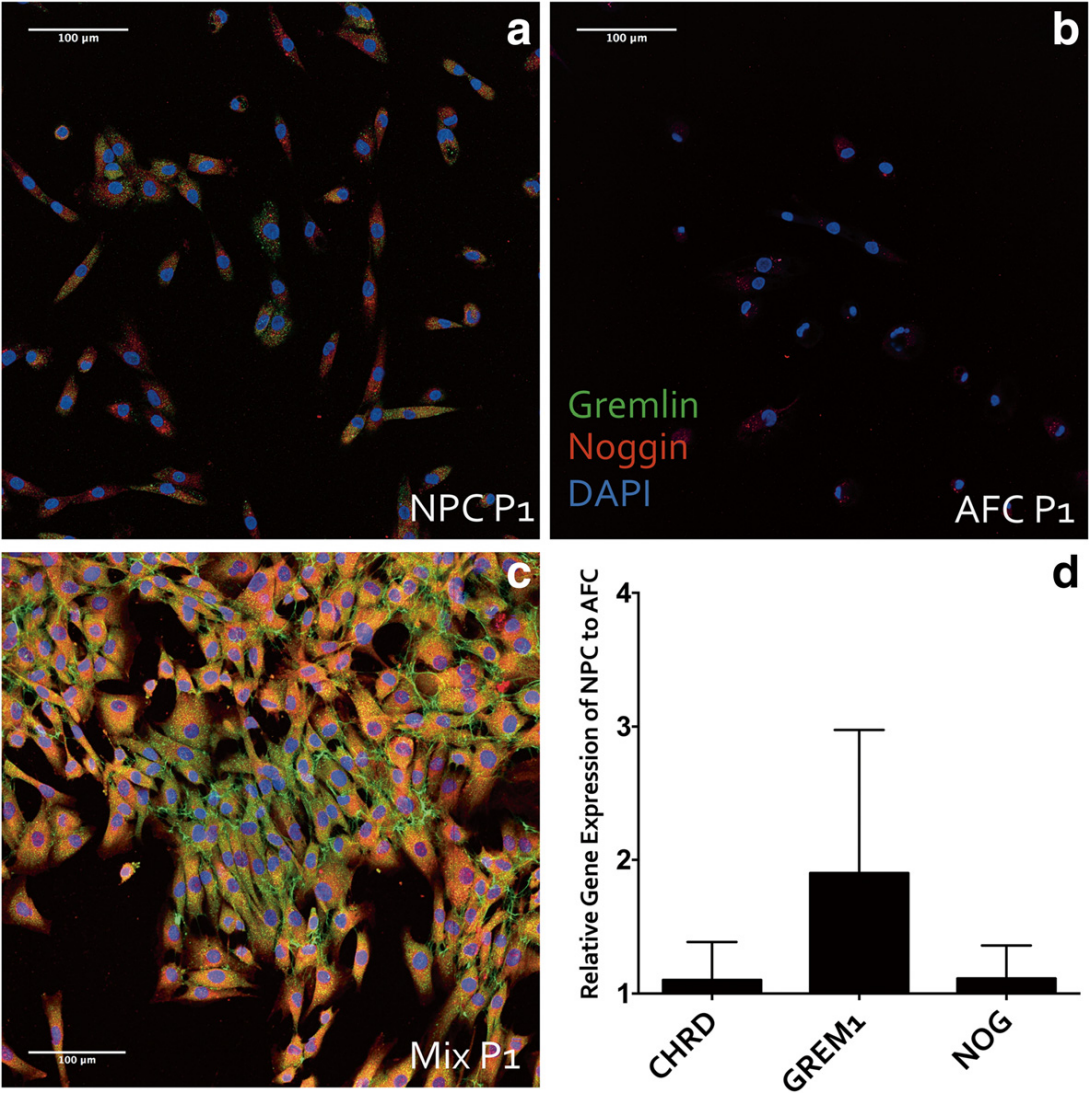
Expression of bone morphogenic protein antagonists Chordin, Gremlin, and Noggin in intervertebral disc (IVD) cells. **a** Immunocytochemistry of GREM1 and NOG in monolayers of passage 1 nucleus pulposus cells (NPCs) (**a**), annulus fibrosus cells (AFCs) (**b**) and (**c**) mixed human IVD cells. GREM1 and NOG were both expressed in NPCs, whereas minimal expression of NOG, but not GREM1, was found in AFCs. GREM1 and NOG were highly expressed in mixed cultures of NPCs and AFCs. **d** Quantification of BMP antagonist gene expression in NPCs relative to AFCs (n = 8 donors) at passage 1. NPC monolayer culture showed no differences in *CHRD* or *NOG* expression compared with NPCs and AFCs. *GREM1* expression was 2-fold higher in NPCs than in AFCs. *DAPI* 4',6-diamidino-2-phenylindole

To investigate the inhibitory effect of IVD cells on osteogenic differentiation of MSCs, the intracellular protein expression of the major BMP antagonists, namely NOG and GREM1, in IVD cells was detected by immunocytochemistry. NOG and GREM1 were both expressed in IVD cells with more intense staining of BMP antagonists in NPCs (Fig. 3a) compared with AFCs (Fig. 3b). The expression of BMP antagonists was highest in a mixed IVD cell population (Fig. 3c).

### Gene expression of DAN family members and Noggin in IVD cells

To gain an insight into which BMP antagonists were responsible for the observed inhibition of osteogenic differentiation, we measured the mRNA levels of major BMP antagonists *CHRD*, *NOG* and *GREM1* by real-time RT-PCR. Gene expression levels were not significantly different between NPCs and AFCs at low passages. However, two-fold higher gene expression of *GREM1* was observed in NPCs compared with AFCs (Fig. 3d). Similarly, *CHRD* and *NOG* gene expression was 1.2-fold higher in NPCs compared with AFCs.

## Discussion

### Implications for spinal non-fusion

Despite being the gold standard for treatment of disc-related back pain, spinal fusion to form a vertebral bone fusion over the disc space has a failure rate of up to 30 % [1, 2]. Although fusion may be improved by different bone graft materials, a clinical study has demonstrated that failure of spinal fusion may be due to the presence of remaining disc tissue that might interfere with the bone union process [3]. Li et al. [4] confirmed this finding using a porcine spinal fusion model in which the fusion rate was only 10 % when the autograft was mixed with NP tissue compared with a fusion rate of up to 70 % when the autograft only was used for fusion. For optimal fusion, the mechanism of non-union must be understood. We therefore investigated the effect of IVD cells on osteogenesis of MSCs to gain an insight into how to improve the ossification process of IVDs.

In this study, we investigated the influence of IVD cells on MSC osteogenesis in a paracrine coculture model. Relative gene expression, ALP activity, and histological analyses showed that IVD cells inhibited osteogenesis of MSCs. The exact mechanism of this inhibition is not fully understood because full mechanistic insights are lacking. We hypothesized that continuous expression of BMP antagonists by IVD cells may interfere with the ossification process during spinal fusion. BMP signaling plays an essential role in skeletal tissue formation and homeostasis, because it induces commitment of MSCs toward osteoblasts [12]. In addition, BMP2 is applied in orthopedics to accelerate bone healing in long bone fractures and spinal fusion. BMP signals are tempered by extracellular signals from BMP antagonists, such as *NOG*, *CHRD*, *GREM1*, and twisted gastrulation (*TWSG1*)*,* which regulate BMP signal transduction [16]. Physiological BMP antagonists negatively regulate BMP ligands [29], and timely regulation of BMPs and BMP antagonists determines healing or non-healing of bone fractures [30]. IVD cells secrete BMP antagonists to regulate formation of the segmental pattern during embryonic development [7, 10]. A microarray study by Minogue et al. [31] indicated a higher gene expression level of *CHRD* in the bovine NP than the AF or chondrocytes.

To delineate the inhibitory effect of IVD cells on MSC osteogenesis, we analyzed the endogenous expression of major BMP antagonists (*CHRD*, *NOG*, and *GREM1*) in IVD cells. We found that BMP antagonists were expressed intracellularly by both human NPCs and AFCs, and were abundant in NPCs. The differential expression of *NOG* and *GREM1* in IVD cells can partly explain the inhibitory effect on the osteogenic differentiation of MSCs. However, it should be noted that, because of insufficient human disc material, the isolated cells were amplified in monolayer culture to compensate for the low cell numbers, and the proteins were detected by immunohistochemistry, which differs from the coculture conditions and their native matrix.

### BMP antagonists

NOG is a major extracellular BMP antagonist that binds to BMPs. It blocks BMP binding to BMP-specific receptors, and therefore negatively regulates BMP-induced osteogenesis [32, 33]. BMPs and NOG appear to be continuously secreted by IVD cells and the entire spine as a potential regulatory mechanism to control bone formation, which is supported by our data and another study in mice [34]. The balance between secretion of BMPs and their antagonists is crucial to maintain normal musculoskeletal functions. Several spinal diseases are caused by dysregulation between bone and IVDs. Diffuse idiopathic skeletal hyperostosis [19, 35] and ankylosing spondylitis (AS) [36] are of particular interest in the context of BMP agonism and antagonism. Both diseases are manifested by excessive bone formation in vertebrae and entheses, although the two diseases presumably have a different pathogenesis [19, 37]. Recently, Xie et al. [38] found that MSCs from patients with AS express higher levels of NOG and BMP2 compared with healthy subjects. MSCs from patients with AS also exhibit a higher osteogenic activity than MSCs from healthy donors [38]. Moreover, it has been demonstrated that *NOG* inhibits migration of human umbilical vein endothelial cells. It has been proposed that notochordal-rich tissue and notochordal cell-conditioned medium [39] contain NOG that might play a crucial role in preventing ingrowth of blood vessels into the healthy disc by downregulation of vascular endothelial growth factor signaling. This observation is in line with our finding of strong positive staining for NOG in NPCs but not in AFCs.

Another BMP antagonist, GREM1, has been recently shown to play a crucial role in a dormant stem cell population that is able to differentiate into cartilage and bone, and even has a reticular stromal potential [40]. GREM1 binds preferentially to BMP2 over BMP4 or BMP7 [41]. Therefore, GREM1 represents a potential therapeutic target to improve spinal fusion therapy, because of the high doses of BMP2 used during spinal fusion surgery. *CHRD* has been reported to be expressed in both bovine and human IVD tissues [17]. The binding of CHRD to BMPs also plays an important role in shaping the BMP morphogenetic field during vertebral development in mouse embryos [8].

Traditionally, the ideal material for fusion has been an iliac crest bone graft. Because of the high rate of postoperative pain and morbidity, bone graft replacement materials with osteoconductive and osteogenic properties, such as demineralized bone matrix, recombinant growth factors, and synthetic implants, have been under extensive investigation. BMP2 has been introduced as one of the most popular alternatives to autologous bone grafts to enhance spinal fusion, but its effectiveness is still the subject of debate [20]. It has been suggested that the osteoconductive activity of the high dose of injected BMPs might be mitigated by cytokines of the DAN family [18] and BMP antagonists such as NOG [16, 21]. A study has shown that an increased BMP concentration induces the expression of BMP antagonists to regulate the amount of osteogenic activity. Therefore, the injected BMP may have a limited ability to induce osteoblast activity because of the intrinsic regulation of BMP activity [42]. Because of the tendency for IVD cells to maintain a balance between BMPs and their antagonists, the action of exogenous BMP2 might be tempered by BMP antagonists secreted from endogenous IVD cells during spinal fusion. Our findings suggest that future therapeutics targeting BMP antagonist signaling pathways might enhance the osteogenic differentiation of MSCs, and therefore the outcome of spinal fusion surgery. Through understanding of regulatory systems and BMP signaling pathways, potential drugs targets may be identified to regulate BMP signals in the process of bone formation [21, 43].

When identifying potential factors to enhance osteogenesis, the binding affinity of BMP antagonists should be considered, because they have different affinities for different BMPs. For example, GREM1 preferentially binds to BMP2, BMP4, and BMP7 [41]. BMP9 may be a more potent osteogenic growth factor than BMP2 because of its resistance to BMP antagonists [44, 45]. BMP9 gene therapy has been shown to improve bone formation during fusion in a rodent model [46]. Intracellular BMP signaling is regulated by the action of inhibitory Smads 6 and 7 that stably bind to type I BMP receptors, thus interfering with Smad-1/5/8 phosphorylation and heterodimerization with Smad4 [43]. On the other hand, the transducer of the ErbB2 gene decreases BMP signaling by binding to type I BMP receptors at the plasma membrane and sequestering Smad-1/5/8. Inactivation of the ErbB2 gene in mice leads to increases in bone formation and the number of osteoblasts [47]. Moreover, blocking BMP antagonists might be an option to enhance osteogenesis [48]. Downregulation of *NOG* by *NOG*-short-hairpin-RNA (shRNA) or small interfering RNA (siRNA) enhances osteogenesis of adipose-derived MSCs [49] and enhances BMP activity in C2C12 cells [50]. *CHRD* knockdown by siRNA also enhances osteogenesis of human MSCs [51]. An *in-silico* study has designed drugs that might interfere with BMP2/NOG binding, but the functions of these drugs still need to be tested [52]. Thus, future experiments should focus on experimental over-expression and/or silencing of specific BMPs and titration of antagonists using clinically relevant primary cells [38].

Our study demonstrated that osteogenesis of bone marrow-derived MSCs is inhibited by IVD cells, and that BMP antagonists are expressed by adult human IVD cells. *GREM1* and *NOG* were more expressed in NPCs than AFCs. At the mRNA level, we found about two-fold higher expression of *GREM1* in NPCs than in AFCs. Our study is limited because we did not check for its expression in the MSC coculture experiment. In addition, other potent BMP antagonists that have been described to play a role in bone homeostasis, such as TWSG-1 and follistatin, should be investigated in more detail in future experiments. In this respect, the role of the chondrocytes in the cartilaginous endplate (CE) should be investigated more deeply in future studies, because the CE cells more frequently remain in the gap during discectomy than NPCs or AFCs.

## Conclusions

In summary, our study provides evidence of inhibition of osteogenic differentiation in MSCs by coculture with IVD cells. The underlying mechanism is unknown, but this effect can be explained partly by the expression of BMP antagonists, namely *NOG* and *GREM1*, by IVD cells when cocultured with MSCs. Targeting these BMP antagonists might be a therapeutic strategy to improve and accelerate the spinal fusion process. In future studies, we will test approaches to inhibit the release of BMP antagonists in vitro and in vivo to induce osteogenic differentiation and improve the outcome of spinal fusion.

## Abbreviations

AFCs: annulus fibrosus cells
ALP: alkaline phosphatase
BMP: bone morphogenic protein
CE: cartilaginous endplate
CHRD: chordin
DAN: differential screening selected gene aberrative in neuroblastoma
FBS: fetal bovine serum
GREM1: gremlin
IVD: intervertebral disc
MSCs: mesenchymal stem cells
NOG: noggin
NPCs: nucleus pulposus cells
PBS: phosphate-buffered saline
TWSG1: twisted gastrulation
